# Identifying the Assembly Configuration and Fluorescence Spectra of Nanoscale Zinc-Tetraphenylporphyrin Aggregates with Scanning Tunneling Microscopy

**DOI:** 10.1038/srep22756

**Published:** 2016-03-07

**Authors:** Xiao-Lei Zhang, Jian-Wei Jiang, Yi-Ting Liu, Shi-Tao Lou, Chun-Lei Gao, Qing-Yuan Jin

**Affiliations:** 1 State Key Laboratory of Precision Spectroscopy, East China Normal University, Shanghai 200062, China; 2 State Key Laboratory of Surface Physics and Department of Physics, Fudan University, Shanghai 200433, China; 3 MOE Engineering Research Center for Nanophotonics and Advanced Instrument, Department of Physics, East China Normal University, Shanghai 200062, China

## Abstract

ZnTPP (Zinc-Tetraphenylporphyrin) is one of the most common nanostructured materials, having high stability and excellent optoelectronic properties. In this paper, the fluorescence features of self-assembled ZnTPP monomers and aggregates on Au(111) surface are investigated in detail on the nanometer scale with scanning tunneling microscopy (STM). The formation of ZnTPP dimers is found in thick layers of a layer-by-layer molecular assembly on Au substrate with its specific molecular arrangement well characterized. Tip-induced luminescence shows a red shift from tilted dimers comparing with the behavior from monomers, which can be attributed to the change of vibrational states due to the intermolecular interaction and the increasing dielectric effect. The nanoscale configuration dependence of electroluminescence is demonstrated to provide a powerful tool aiding the design of functional molecular photoelectric devices.

Porphyrin and metalloporphyrin molecules have attracted a lot of attention because of their well-known optical, electronic, and biological properties. The self-assembled aggregates of these molecules produced under the influence of electrostatic and Van der Waals forces are used as building blocks^1^,^2^ to construct artificial functional structures with wide optical related application in biomimetic photosynthesis^3^,^4^,^5^, solar cells^6^,^7^,^8^, nonlinear optical systems^9^,^10^,^11^, and organic photovoltaic devices^12^,^13^,^14^. For example, as a widely available porphyrin, ZnTPP (zinc-tetraphenylporphyrin) and its modified oligomer can define excellent long-wavelength-emitting platforms to fabricate the near infrared (NIR) light-emitting diodes (LEDs)^15^. It is thus extremely important to understand the relationship between the structural and optical properties of the porphyrin aggregates. However, most studies in this field were carried out in solution^16^,^17^,^18^ which gives the collective optical behavior but can not directly link to the specific structures. In contrast to the use of a very low concentration solution for ensemble average measurement by laser, thin molecular films on a substrate should provide a stable and local environment to study the nanoscale structure and related optical properties for understanding the essential physical and chemical interaction. In recent years, by electronically decoupling the molecules from the substrate using insulated spacers^19^,^20^,^21^, molecular multilayer^22^,^23^,^24^,^25^ and self-decoupled molecules^26^,^27^,^28^, the fluorescence feature of molecular monomer has already been successfully investigated on the nanoscale with STM-induced luminescence (STML). Importantly, STML offers the possibility of directly connecting the optical transition with the structural arrangement of the molecules. Nevertheless, studies on the light emission and configuration dependence of nanoscale porphyrin aggregates are still limited. Understanding the porphyrin assembling process at the molecular level on substrate is crucial to synthesis basic units of functional structures. Utilizing the variation from molecular monomer to aggregates, the fluorescence emission energy could be predictably changed to broaden the wavelength regime. It is of importance to tune the local assembled and spectroscopic properties for understanding the aggregation phenomena and the energy transfer in tunnel junction, and then meeting some specific optoelectronic requirements. The configuration change process from monomers to aggregates and its influence on the luminescence is not very clear, but obviously important for designing multi-band photoelectric devices and exploring the mechanism of molecular energy level transition.

In this work, we investigate the self-assembled growth of multilayer ZnTPP molecules formed on Au(111) surface which is widely used for porphyrin monolayers^2^,^25^ and study their optical properties layer by layer. The structure and optical properties of ultrathin molecular films have been found to become complicated with different layers, which can include flat-lying monomers, irregular clusters, tilted monomers and dimers. All of the featured structures can give relevant, well-characterized spectra in the nanoscale tunneling junction. Red-shift and the enhancement of fluorescence, which were obtained on the tilted dimers array at the nanoscale, are attributed to the intermolecular interaction of the dominant molecular dimers and the increasing effective dielectric constant of the tunnel junction.

## Results and Discussion

**Figure 1:**
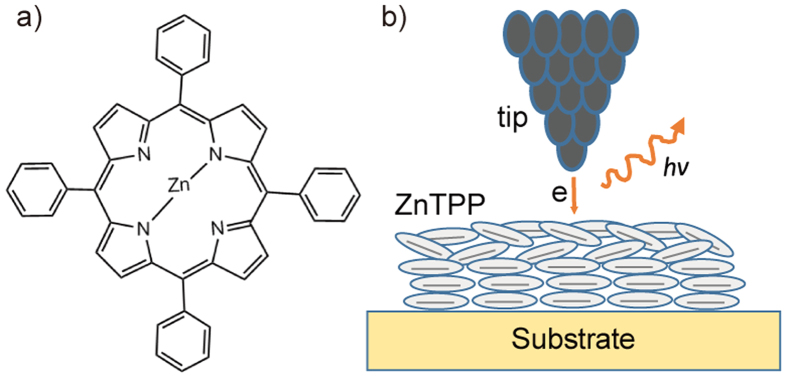
(**a**) Chemical formular of ZnTPP. (**b**) Schematic of tip-molecules-substrate junction and local tunneling electron induced luminescence.

ZnTPP comprises a planar porphyrin core with a metallic centre and four phenyl substituents (illustrated in Fig. 1a). As schematically shown in Fig. 1b, ZnTPP can be adsorbed on the Au(111) substrate in a layer-by-layer configuration. It has been proved that the top-layer porphyrin molecules would emit photons governed by resonant nanocavity plasmons (NCPs) in a highly confined STM nanocavity^25^ with the bottom molecules acting as decoupling spacer.

**Figure 2:**
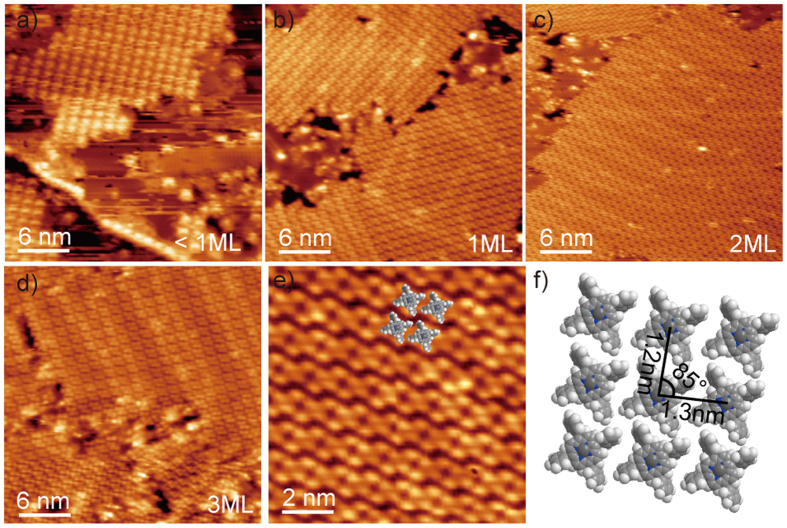
(**a–d**) STM images (30 × 30 nm^2^) of the layer-by-layer growth of ZnTPP molecular structures of sub-monolayer (+1.5 V, 20 pA), 1 ML(+2.5 V, 20 pA), 2 ML(+2.8 V, 10 pA) and 3 ML (+2.8 V, 10 pA). (**e**) High-resolution STM image of ZnTPP molecules (10 × 10 nm^2^, + 2.8 V, 10 pA). (**f**) tentative model of the molecular parallel arrangement.

Figure 2 (a–d) show a ZnTPP molecular assembly on Au(111) at layer thicknesses varying from sub-monolayer (ML) to 3 ML. The flat Au terraces are gradually covered with highly ordered ZnTPP molecular arrays and the surface-reconstructed ‘herringbone’ pattern of the Au substrate is still clearly observed. The high-resolution STM image (Fig. 2e, 10 × 10 nm^2^) shows that each individual ZnTPP molecule can be clearly recognized as four bright lobes (phenyl moieties) surrounding a dark spot (zinc ion). The equal brightness of the lobes suggests that the porphyrin core is oriented parallel to the surface of Au(111). It is generally believed that the intermolecular forces are the main driving force to facilitate the formation of stable planar molecular arrangements since the van der Waals forces between molecules and substrate are rather weak. A tentative structural model of the molecular parallel arrangement is shown in Fig. 2f. The intermolecular distances are ~1.3 nm and ~1.2 nm for two directions at an angle of ~85°.

**Figure 3:**
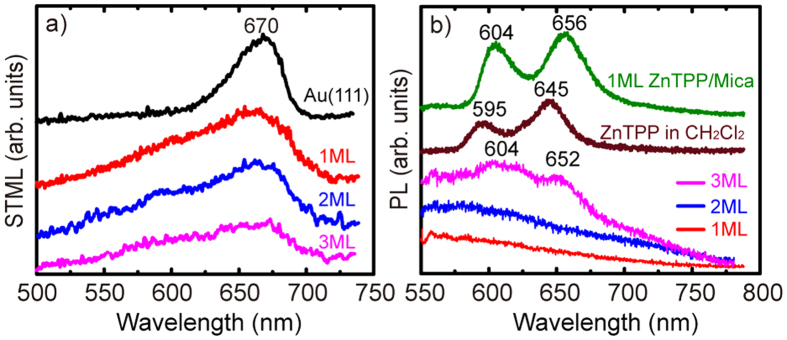
(**a**) STML spectra for the pristine Au and ZnTPP/Au at 1–3 ML. (**b**) PL spectra for ZnTPP in CH_2_Cl_2_ solution, 1 ML ZnTPP/Mica and 1–3 ML ZnTPP/Au.

Figure 3a gives the STML spectra from 1 to 3 ML ZnTPP on Au(111). They show a single-peak feature around 670 nm, the peak position of the light emission from a pure Au substrate. Based on previous reports^22^,^25^, the molecule-modified emission peak is still related to tip-induced plasmons (TIPs) and molecular fluorescence is quenched due to the molecule-substrate interaction because of the nonradiative energy transfer from excited molecules to the substrate. The electronic decoupling effect of 3 ML thickness is not enough to see the molecular fluorescence in STML. However, as shown in Fig. 3b, the photoluminescence (PL) spectrum of 3 ML ZnTPP sample has two small bumps at ~604 and 652 nm, which is in agreement with the PL spectrum of ZnTPP monomers in CH_2_Cl_2_ solution or that of 1ML ZnTPP film on Mica where there is no quenching effect from the metallic substrate. ZnTPP molecules are firstly excited to the electronic singlet state S_1_ and higher excited singlet S_2_. Then the electrons on the excited states S_1_ and S_2_ usually prefer non-radiative decay to the lowest excited vibrational state S_1_(0) by rapid internal relaxation and radiative decay to the electronic ground state S_0_ with light emission. So the double-peak characteristic originates from the zero-phonon emission Q_x_(0, 0) and the one-phonon emission Q_x_(0, 1) because of the π^*^-π transitions of porphyrins, associating with the HOMO-LUMO (highest occupied molecular orbital-lowest unoccupied molecular orbital) gap of the molecule^29^. The 604 and 652 nm features are attributed to normal decay channels of excited states from S_1_(0) to the two electronic ground states S_0_(0) and S_0_(1), respectively. There are two main reasons behind the difference of STML and PL spectra for 3 ML ZnTPP film. The reasons are firstly that the tunneling current is very local compared with the laser spot, and secondly that the optical coupling efficiency between the parallel molecular dipole and the perpendicular exciting field in the tip-substrate cavity is weak.

**Figure 4:**
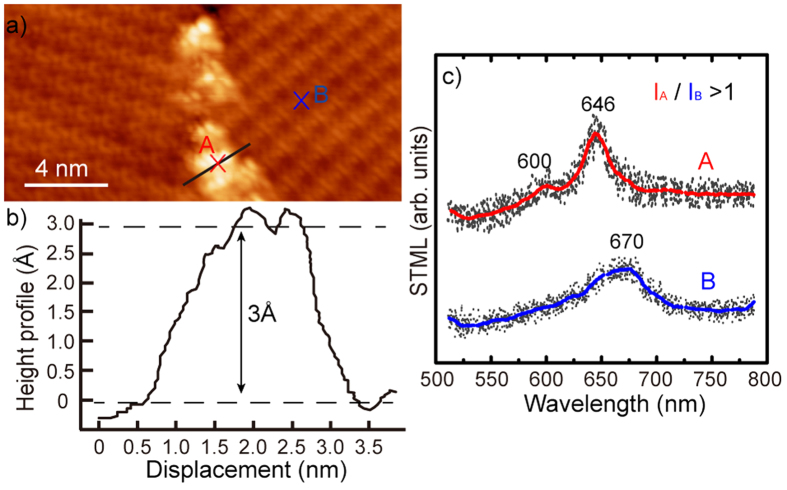
(**a**) STM image of ZnTPP cluster at the island boundary (30 × 10 nm^2^, +2.5 V, 10 pA). (**b**) Height profile for the line trace across the cluster in (**a**). (**c**) STML spectra on the cluster (A) and flat domain (B).

As we increase the coverage to 4 ML, the ZnTPP molecules still possess the flat-lying orientation and the intrinsic molecular electroluminescence remains absent on the 4 ML ZnTPP film. However, some configurational changes are observed at the island boundary, for example molecular chains as shown in Fig. 4a. With increasing thickness, the substrate-molecule perpendicular interaction becomes weak, which may cause a change of the molecular arrangement. From the height profile in Fig. 4b for the line trace across the cluster in (a), the chain has a lateral size of about 3 nm and a height of about 3 Å, which seems not to be one single molecule but a molecular cluster. STML measurements were taken on the ordered domain (region B) and the cluster (region A), and their STML spectra are shown in Fig. 4c. We find that the emission intensity from the cluster (averaged as the red curve) is higher than that from the domain (blue curve), indicating the possible emergence of molecular luminescence for clusters. The spectrum from position B shows a single-peak feature around 670 nm, which is characteristic of plasmon-mediated emission from the Au(111) substrate (Fig. 3a), while that from position A exhibits two characteristic molecular peaks around 600 and 646 nm, similar to the PL peaks of ZnTPP monomers in CH_2_Cl_2_ solution. The increasing intensity and the two-peak feature at position A demonstrate that the fluorescence from clusters at the island boundary of 4 ML ZnTPP film includes luminescence contributions from molecules. In addition to the decoupling effect, the axial dipole oscillation with NCPs is crucial to the generation of molecular fluorescence^25^,^30^,^31^,^32^. The above spectral information suggests that the molecular core plane in the cluster may not be parallel to the substrate and thus contributes axial dipole orientation.

**Figure 5:**
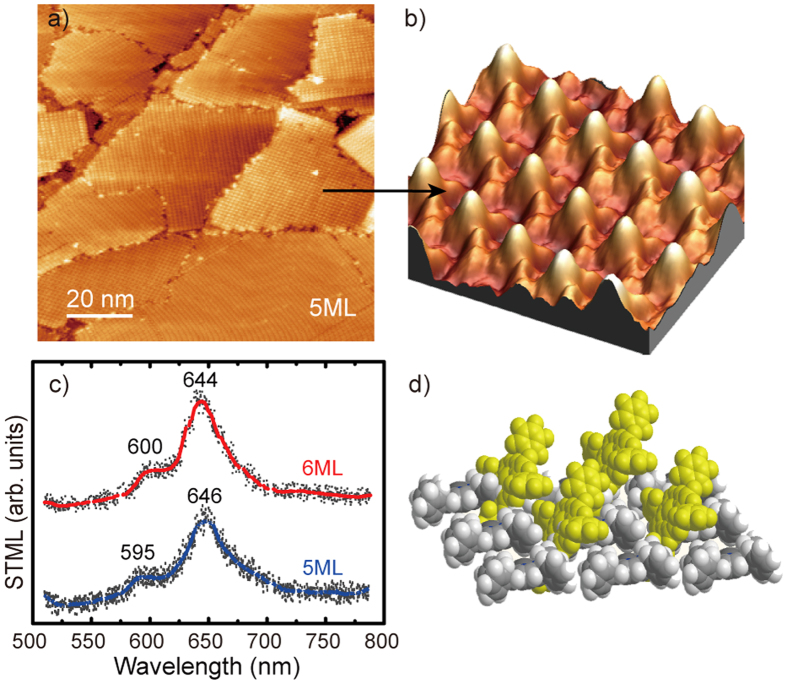
(**a**) STM image (100 × 100 nm^2^, + 2.6 V, 10 pA) and (**b**) high-resolution (5 × 5 nm^2^, + 2.6 V, 10 pA) 3D-view STM image of 5 ML ZnTPP/Au. (**c**) STML spectra on 5 and 6 ML ZnTPP/Au. (**d**) tentative model of the molecular tilted arrangement.

When the coverage is increased to 5 ML, the molecules are increasingly tightly packed with the consequence that the increasing compression force between molecules obviously leads to the modification of the molecular stacking configuration. As a result, the non-parallel orientation of the molecule core plane to the substrate is clearly seen on the 5 ML film. Figure 5a,b are, respectively, the STM image and high-resolution 3D-view STM image for the 5 ML ZnTPP film. The asymmetric feature of each ZnTPP molecule is widely observed with one lobe brighter than others (~1.3 Å higher than other lobes) in a four-lobe pattern. Molecular double-peak luminescence, with the emission wavelength centered around 600 and 646 nm, can be easily detected from the islands of 5 and 6 ML molecules (shown in Fig. 5c). The typical peak of 670 nm from Au substrate no longer exists. Based on the configuration and spectrum characteristic, each molecule of the top monolayer is relatively tilted with one phenyl substituent on the upper side, as shown in Fig. 5d. The tilted molecular alignment may give additional perpendicular dipole components and benefit the resonance enhancement of plasmon-coupled emission.

**Figure 6:**
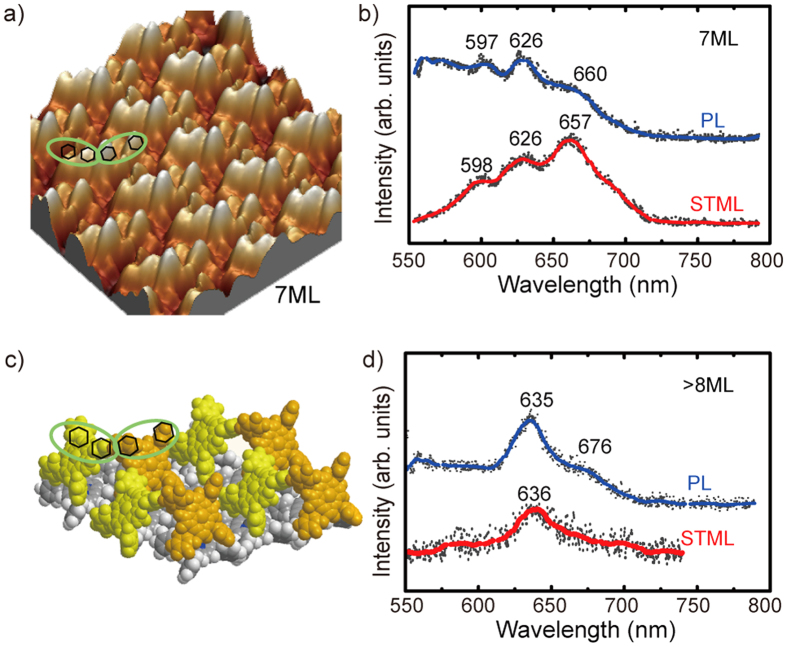
(**a**) High-resolution (5 × 5 nm^2^, + 2.8 V, 10 pA) 3D-view STM image of titled molecular dimers of 7 ML ZnTPP/Au. (**b**) PL and STML spectra on the molecular dimers of 7 ML ZnTPP/Au. (**c**) tentative model of the titled dimers. (**d**) PL and STML spectra on ZnTPP film at more than 8 ML.

In contrast with the 5th monolayer of the ZnTPP molecular film, the one-brighter-lobe feature shown in high-resolution STM image has changed, showing some aggregation in molecular islands at the 7th monolayer. Figure 6a shows a high-resolution 3D-view STM image. Two brighter lobes (~0.8 Å higher than the others) for each molecule begin to emerge and the two neighboring molecules seem to be paired in a certain direction like two wings. The corresponding STML and PL spectra are given in Fig. 6b. Three-peak STML spectrum is acquired over the unusual molecular alignment of 7 ML ZnTPP/Au, substantially same as the *in-situ* PL spectrum (in the same external conditions as STML, described in the below “Methods”). In addition to the monomer emission band around 600 and 660 nm, a new emission band around 626 nm starts to emerge. The red-shift for Q_x_(0, 1) band from 646 nm at 5 ML to 660 nm at 7 ML may have two origins. One contribution results from the fact that increasing molecules will cause more and more compact arrangement, and enhancive nonradiative energy transfer between molecules including monomers and aggregates may decrease the emission energy of the S_1_-S_0_ transition. The second effect contributing to the shift is the increase of the effective dielectric constant of the tunnel junction with increasing molecular layers, which will weaken the axial dipoles’ polarization mediated by NCPs. The new fluorescence emission at 626 nm may rise from the Q_x_(0, 0) emission of ZnTPP dimers by consideration of the aggregated tilted configuration illustrated by the model shown in Fig. 6c. The upper two phenyl rings of a single tilted molecule are marked by small hexagons in an ellipse according to the arrangement in Fig. 6a. It should be emphasized that although the STML focused on the highly localized area, the current of 1nA will cause the tunneling region not confined to the top dimer island and the below monomer layer also contributes to the emission. As a result, the STML spectra demonstrate the coexistence of porphyrin monomers and dimers with 3 peaks similar to the PL spectra in the 7th monolayer. In addition to π-π stacking interactions, some chemical bonding may occur in the close-packed tilted molecules leading consequently to the pairing effect. Comparing the Q_x_(0, 0) emission of ZnTPP dimer at ~626 nm with monomer at ~600 nm indicates that dimer’s HOMO-LUMO gap is narrower than that of the monomer because of the energy exchange and dissipation between two molecules in a dimer. Moreover, as shown in Fig. 6d, on increasing the thickness to greater than 8 ML, the two peaks of monomer emission around 600 and 660 nm almost disappear and instead the dimer fluorescence excited by tip or laser is dominant with a red-shift Q_x_(0, 0) band around 636 nm from previous 626 nm. In addition, the Q_x_(0, 1) emission of dimers begins to appear with a small feature around 676 nm (see PL data in Fig. 6d). Because of the “dirty” tip adhered molecules after scanning on thick layers and the increased tip-metal separation, the STML signal here is so weak that it is very difficult to distinguish the 676 nm peek from the back ground noise. Although it is difficult to get clear STM images on the thicker films, the characteristics of the spectrum indicate that the porphyrin dimers become the main components for ZnTPP films of thickness greater than 8 ML. Increasingly packed ZnTPP dimers lead to potential energy transitions among them with consequent spectral profile modification. The observed red-shift emission peak from 626 to 636 nm (Fig. 6b,d) exhibits a slight narrowing of HOMO-LUMO gap for the ZnTPP dimers. Besides the increasing dielectric effect, the origin of such a red-shift phenomenon is associated with the changing vibrational states of the aggregated molecules. The vibrational states of both the excited singlet state S_1_ and the singlet ground state S_0_ for dimers might be expected to change, causing the nonradiative energy transfer between neighboring molecules and dissipation with red-shifted light emission.

Two major mechanisms, hot-electron injection and NCPs-mediated excitation, are responsible for the generation of molecular excited states in a tip-molecules-substrate junction, as reported previously^25^. The excited molecules then decay to the ground state via Frank-Condon transitions with fluorescence emission. It should be pointed out that, with the increase of the molecule film thickness, the molecular arrangement begins to change and the interaction among the molecules becomes complicated. As a result, the ground and excited states of the molecules could be modulated with subsequent broadening and enhanced spectral region.

## Conclusion

We have successfully observed the light emission from both metalloporphyrin monomers and aggregates in STM nanoscale junction, identified by *in situ* comparison with the laser-induced photoluminescence. Increasing the thickness of molecular monolayers was found to change the parallel molecular configuration, inducing substantial tilt, and to generate molecular monomer fluorescence. Moreover, we observed the red-shift dimer fluorescence of tilted aggregated molecules by continuously increasing molecular layers with the changes of the molecular vibrational states. These findings may be used to design specific molecular configurations to obtain tunable light and to quantify the changes of molecular energy level.

## Methods

Our experiments were carried out in an ultrahigh-vacuum (UHV), low-temperature STM system (Createc Inc.) with a base pressure of ~1 × 10^−10^ mbar. All STM images were taken in the constant current topographic mode with sample biased at a sample temperature of ∼80 K. The atomically clean Au(111) single-crystal substrate was prepared by several cycles of ion sputtering and annealing process. ZnTPP films were thermally evaporated onto Au(111) substrate at room temperature. The deposition rate is ~0.1 ML/min, which has been monitored by a Constant Voltage and Current DC Power Supply and calibrated by STM images. The layer-by-layer growth of ZnTPP molecules on Au was confirmed by STM imaging. Pt-Ir tips used for imaging and photon emission were cleaned by argon ion sputtering in UHV and the constant NCP mode was modified by voltage pulses on clean metal surfaces. The light emission from the tunneling junction acquired at ∼80 K was collected by a two-lens alignment (one near the tip-sample gap region and the other located outside a viewport of the UHV system) with an optical fiber connected to the highly sensitive photon detection system, a liquid nitrogen cooled charge coupled device (CCD) spectrometer (Princeton Instruments). The thermal drift at ∼80 K is ∼10 pm/min. In order to repeat our measurements, we first modified the tip condition on the clean Au(111) surface by voltage pulses and then the same tip was used to image the molecular surface at low currents (typically 10pA). After that, the tip was statically positioned on the certain area to excite light emission. The STML spectra were obtained in the tunneling regime typically at 2.8 V and 1 nA over 30 mins and were not corrected for the wavelength dependent sensitivity of the detection system.

## Additional Information

**How to cite this article**: Zhang, X.-L. *et al.* Identifying the Assembly Configuration and Fluorescence Spectra of Nanoscale Zinc-Tetraphenylporphyrin Aggregates with Scanning Tunneling Microscopy. *Sci. Rep.* **6**, 22756; doi: 10.1038/srep22756 (2016).
